# Manufacture and characterisation of EmDerm—novel hierarchically structured bio-active scaffolds for tissue regeneration

**DOI:** 10.1007/s10856-018-6060-6

**Published:** 2018-06-05

**Authors:** Xuxin Lim, Matthew Potter, Zhanfeng Cui, Julian F. Dye

**Affiliations:** 1 Tissue Engineering Group, Institute of Biomedical Engineering, Department of Engineering Science, University of Oxford, ORCRB, Roosevelt Drive, Headington, Oxford, OX3 7DQ UK; 2 Department of Plastic Surgery, Oxford University Hospitals NHS Foundation Trust, West Wing, John Radcliffe Hospital, Headington, Oxford, OX9 9DU UK

## Abstract

There are significant challenges for using emulsion templating as a method of manufacturing macro-porous protein scaffolds. Issues include protein denaturation by adsorption at hydrophobic interfaces, emulsion instability, oil droplet and surfactant removal after protein gelation, and compatible cross-linking methods. We investigated an oil-in-water macro-emulsion stabilised with a surfactant blend, as a template for manufacturing protein-based nano-structured bio-intelligent scaffolds (EmDerm) with tuneable micro-scale porosity for tissue regeneration. Prototype EmDerm scaffolds were made using either collagen, through thermal gelation, fibrin, through enzymatic coagulation or collagen-fibrin composite. Pore size was controlled via surfactant-to-oil phase ratio. Scaffolds were crosslink-stabilised with EDC/NHS for varying durations. Scaffold micro-architecture and porosity were characterised with SEM, and mechanical properties by tensiometry. Hydrolytic and proteolytic degradation profiles were quantified by mass decrease over time. Human dermal fibroblasts, endothelial cells and bone marrow derived mesenchymal stem cells were used to investigate cytotoxicity and cell proliferation within each scaffold. EmDerm scaffolds showed nano-scale based hierarchical structures, with mean pore diameters ranging from 40–100 microns. The Young’s modulus range was 1.1–2.9 MPa, and ultimate tensile strength was 4–16 MPa. Degradation rate was related to cross-linking duration. Each EmDerm scaffold supported excellent cell ingress and proliferation compared to the reference materials Integra™ and Matriderm™. Emulsion templating is a novel rapid method of fabricating nano-structured fibrous protein scaffolds with micro-scale pore dimensions. These scaffolds hold promising clinical potential for regeneration of the dermis and other soft tissues, e.g., for burns or chronic wound therapies.

## Introduction

Hierarchical interconnected porous architecture and nano-scale structure are fundamental requirements of three-dimensional protein-based bio-active scaffolds, which are essential for the functions of cell conductivity, nutrient perfusion, angiogenesis and vasculogenic differentiation [1–3]. However, there is a need for effective, controllable, scalable methods of producing such nanostructured scaffolds.

One method for achieving controlled porosity in protein hydrogels is controlled freezing and lyophilisation, in which pores are formed by material exclusion from the ice crystal porogen. This typically results in dense lamellar structured material, largely devoid of nano-scale structure. This is shown by many of the current scaffolds, notably acellular collagen scaffolds [4]. This method is still in widespread use despite the long processing times required to make such scaffolds [5]. Cellularisation and vascularisation into such materials is relatively slow [6]. Foam formation by aeration is another methodology [7–10], but also has some limitations, due to a large exposed air interface for protein denaturation, intrinsic bubble instability and foam drainage during gelation and cross-linking, which cause difficulty in achieving a biologically acceptable degree of homogeneity.

Much recent attention has been focussed on the powerful and highly controllable bottom-up manufacture methodologies [3, 11]. Electrospinning is an established method of forming micro and nano-scale fibre meshes, but is not amenable to manufacturing structures at the thickness and consistency for the commercial scale-up required for marketing three-dimensional scaffolds. While 3D-printing and rapid-prototyping are emerging technologies which are able to create soft scaffold structures, the concept of building macroscale structure from a nano-scaled filament at the scale required for commercial manufacture, remains challenging. Therefore, new methods of controllable rapid and versatile manufacture of nano-structured regenerative biomaterials would represent a significant advance in healthcare technology.

Emulsion templating, a form of ‘polymerisation of high internal phase emulsion’ systems (polyHIPEs), is a well-established method of manufacturing nano-porous polymer membranes e.g., for water purification films, catalytic panels, controlled release storage and tissue regeneration [12]. Its potential benefits as a method of manufacturing porous scaffolds for tissue engineering are the ease of control of porosity by controlling emulsion droplets size, relatively rapidity structure formation in bulk, and cost-effectiveness. Its use with biological polymers has so far been limited to polysaccharides [13] and the denatured polypeptide, gelatin [14] and not with native proteins such as collagen and fibrin.

There are several major limitations to emulsion templating of protein structures: protein denaturation (by adsorption at hydrophobic interfaces and by surfactant agents), emulsion instability, complexity of introducing stabilisation methods, and elution of the oil-phase and surfactant [15]. Proteins typically adsorb to and denature at oil-water interfaces [16–18] so the creation of very large surface area to volume ratio interfaces (in the order of 500 cm^2^/ml), and short diffusion distances (in the range 1–10 μm) necessarily presents a system that will favour any denaturation process. Moreover, the use of surfactants needed to form a stable emulsion will also be likely to adsorb onto and denature proteins [19]. Additionally, any process of emulsion ripening or maturation, will counteract the desired stability of the emulsion droplets as a template. Further issues of particular consideration for protein scaffolds concern post-formation stabilisation, for example by a compatible chemical cross-linking method, and elution of the oil phase without disruption of the formed scaffold. For example, phase separation involves release of significant energy, which is capable of disrupting the formed material as the oil phase separates. All these considerations determine the feasibility and resultant biocompatibility of the scaffold produced.

The underlying hypothesis we investigate is that a surfactant hydrophilic boundary layer that prevents protein adsorption and denaturation at the emulsion interface will enable protein-based three-dimensional hydrogels to be formed into porous scaffold structures with minimal denaturation. We investigate this by addressing each of the issues required to establish a feasible manufacture process. A biocompatible emulsion has been established in our lab using non-ionic surfactants to control emulsion droplet size, prevent denaturation of proteins in the aqueous phase of emulsion, and allow successful formation of protein scaffolds. Almost all commercially available skin substitutes are made of collagen or collagen composites [20] and fibrin is also widely used as an extracellular matrix [21]. Therefore, we have used these proteins as scaffold materials, to evaluate the influence of protein type on biocompatibility of emulsion templated dermal scaffolds (EmDerm). Their efficacy in supporting cell proliferation and migration are compared to commercially the available scaffolds Matriderm^TM^ and Integra^TM^.

## Methods

### Materials

Reagents were obtained from Sigma Aldrich, Poole, UK unless otherwise stated. Proteins used for scaffolds were type I rat tail collagen 5 mg/ml in acetic acid (First Link, Wolverhampton, UK), bovine fibrin and bovine thrombin. 2-(N-morpholino) ethanesulfonic acid (MES) biochemical grade (Fisher Scientific, Loughborough, UK) and sodium chloride were used for the buffer. Decane, Tween 20 and Span 20 were used for emulsions. 1-ethyl-3-(-3-dimethylaminopropyl) carbodiimide hydrochloride (EDC) and sulfo-*N*-hydroxysuccinimide (NHS), pure ethanol and isopropanol were used for cross-linking and oil elution. The excipients used were polyvinyl alcohol (PVA) (99% hydrolysed, MW 89,000 to 98,000), polyethylene glycol (PEG) (MW 6000), Pluronic F-68 (P68) and mannitol (M). 0.25% Trypsin-EDTA solution for cell culture was used for stability testing and the Cell Counting Kit (CCK-8) assay was used to measure cell proliferation.

### Manufacturing of scaffolds

Macro-emulsion mixtures comprising of decane, Span 20/Tween 20 at a calculated HLB of 13 and an aqueous buffer (25 mM MES, 150 mM NaCl pH 7.4) were mixed in a 60 ml syringe with the tip removed, for 15 seconds at 1000 rpm using a high-speed mixer (Ceframo BDC6015, Ontario, Can). Emulsions with 0.1, 0.3, 0.5 and 0.7% surfactant concentration were prepared. To manufacture scaffolds from each protein type, each of the emulsions was added to each protein solution designated as follows: collagen (Col), fibrinogen plus thrombin (Fbn) or a mixture of collagen and fibrin solution at a 1:1 volume ratio (ColFbn). The gelling solution of type-I collagen was used at 4.5 mg/ml, fibrinogen was prepared at 20 mg/ml and thrombin at 10 units/ml in in 25 mM MES/150 mM NaCl (pH 7.4) buffer. Each emulsion-scaffold was left to gel at 37 °C for 30 min and then cross-linked with EDC:NHS (21.9:8.68 mM, 5:2 molar ratio) in 80% ethanol [22]. The scaffolds were then washed in 80% isopropanol, then deionized water three times for 15 minutes. At this point scaffolds were further incubated with a 1% w/v excipient solution (e.g. P68, PVA, PEG or M). Collagen scaffolds were freeze-dried at −30 °C and fibrin scaffolds were freeze-dried at −40 °C.

The possible effects of the different excipients used, on functional parameters and scaffold morphology, were compared using scaffolds made at large pore size with 0.1% surfactant concentration. The effect of surfactant concentration was compared between scaffolds made using F68 as the excipient.

### Pore size of scaffolds

Scaffolds were cut using a scalpel so that the cross-section of each scaffold was lying horizontally on the carbon tape mounted on the aluminium stubs for scanning electron microscopy (SEM) imaging. Three images were taken per scaffold and the diameters of 20 random pores were averaged out to calculate estimated mean pore size of each scaffold. Scaffold morphology (e.g. fibrous or smooth) was also noted.

### Mechanical properties of scaffold

Scaffolds that were cross-linked for an hour, with different excipients and surfactant concentrations, were tested for mechanical properties (Instron 5582 UTM). Each scaffold was cut into strips measuring 2 mm × 40 mm × T where T is the variable thickness of each scaffold. Each end of the strips were anchored into the jaws of the Deben tensile testing machine and stretched until the scaffold broke into two pieces. Young’s modulus and ultimate tensile strength (UTS) were calculated for each scaffold using stress-strain curves obtained. Each test was performed in triplicate.

### Degradation profile of scaffold

Scaffolds were cut into 6 mm discs using a punch biopsy and incubated in PBS for 7, 14, 21 28 and 35 days. The scaffolds were freeze-dried after each time point and dry mass of each scaffold was recorded. We introduced an enzymatic proteolysis assay to compare the degradation profiles of each scaffold, over a relatively short period. Scaffold discs were incubated in 0.25% (1×) Trypsin solution in PBS, which was changed daily for up to 7 days. Individual discs were freeze-dried at the following time points: Days 1, 3, 5 and 7 and dry mass of each scaffold was measured. Each test was performed in triplicate.

### Cell culture

Human dermal fibroblasts derived from neonatal foreskin (HDF) were obtained from Invitrogen, UK. They were cultured in standard Dulbecco’s Modified Eagle Medium (DMEM) media (Gibco, ThermoFisher Scientific, UK) with 20% foetal bovine serum and 1% penicillin/streptomycin. Only cells from Passage 2 to 9 were used in the experiment. Human dermal microvascular endothelial cells derived from neonatal foreskin that have been hTERT immortalized (HDE), were obtained from ATCC, UK. They were cultured using Vascular Cell Basal Medium with Microvascular Endothelial Cell Growth Kit-VEGF supplement containing: rhVEGF (5 ng/ml), rhEGF (5 ng/ml), rhFGF basic (5 ng/ml), rhIGF-1 (15 ng/ml), L-glutamine (10 mM), heparin sulfate (0.75 Units/ml), hydrocortisone (1 µg/ml), ascorbic acid (50 µg/ml) and foetal bovine serum (5%). Human bone marrow mesenchymal stem cells transfected with GFP (MCS) were obtained courtesy of Dr James Li (Hong Kong University) and cultured in DMEM media.

### Cytocompatibility of scaffolds

Scaffolds were cultured with each cell type to investigate cytocompatibility. Each scaffold was cut into 6 mm discs using a punch biopsy and washed with PBS three times before incubating overnight in media. On the following day, each scaffold was seeded with 5000 cells/well and left overnight to allow cells to adhere. On the next day, the seeded scaffolds were transferred into a new well plate and allowed to incubate for up to 14 days. Media was changed on alternate days. On days 3, 7 and 14, cell growth in each scaffold was measured with the CCK-8 assay (2-(2-methoxy-4-nitrophenyl)-3-(4-nitrophenyl)-5-(2,4-disulfophenyl)-2H-tetrazolium, monosodium salt (WST-8) plus 1-methoxy phenazine methosulfate (PMS) reagent (WST8/PMS)) (Sigma, UK). Briefly, 10 μl of WST8/PMS reagent was added to 100  μl of fresh media in each well and incubated for 4h at 37°C. 50 μl of the resulting solution was pipetted into a new plate for colorimetric assay of the reduced formazan product at 450 nm. Each test was done in triplicate. On day 14, the scaffolds were fixed in normal buffered formalin for microscopy.

### Wide-field imaging of seeded scaffolds

Each of the seeded scaffolds was stained using fluorescent antibodies for microscopy. Scaffolds seeded with human dermal fibroblasts were stained with 1:6 phalloidin (Alexa Fluor 488, Thermo Fisher) and scaffolds seeded with human dermal endothelial cells were stained with 1:250 mouse anti-human CD31 (Dako) and 1:1000 rabbit anti-human vWF (Dako). Secondary staining was done using goat anti-mouse (Alexa Fluor 488, Thermo Fisher) and goat anti-rabbit (Alexa Fluor 568, Thermo Fischer). Scaffolds seeded with mesenchymal stem cells were not stained, since they expressed GFP. Z-stacks were taken to visualize cell migration through the scaffold by wide-field imaging at 20× magnification. Images were deconvoluted using AutoQuant X3 (Media Cybernetics) and visualised using Bitplane (Imaris software, Version 9.1).

### Statistical analysis

Data between different scaffolds and time-points were analysed using one-way analysis of variance (ANOVA) with Tukey’s post-hoc multiple comparisons tests to evaluate statistical significance. All statistical analysis was performed using GraphPad Prism Version 4.0 for Windows. A p-value of less than 0.05 is considered to be statistically significant. Where applicable, * denotes a p–value of <0.05, ** denotes a p–value of <0.01 and *** denotes a p-value of <0.001.

## Results

### Scaffold structure and porosity

**Fig. 1:**
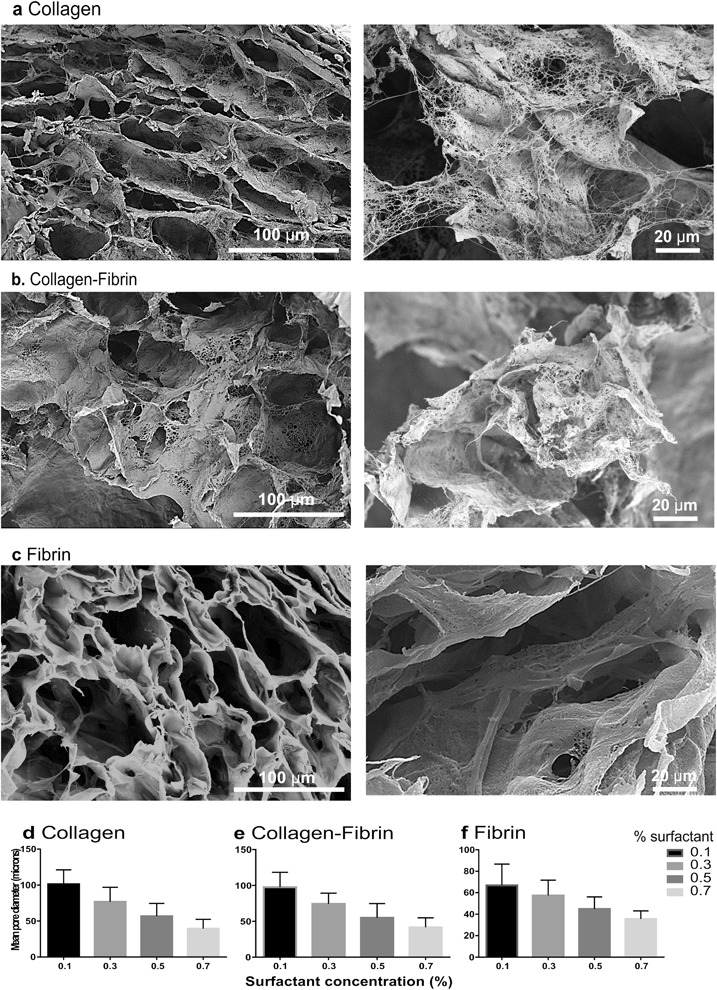
Structure of EmDerm scaffolds formed with 0.1% surfactant from: Collagen (**a**), Collagen-Fibrin (**b**) or Fibrin (**c**). SEM micrographs of representative fields of scaffolds show meso-scale structure and pore size (1000×) and nano-scale structure (2000×). The effect of surfactant concentration, from 0.1 to 0.7%, in the mixed phase templating system on the distribution of pore diameters (mean ± SD) of each type of EmDerm scaffold, collagen (**d**), collagen-fibrin (**e**) and fibrin scaffolds (**f**)

The use of decane oil-in-water (o/w) emulsions was successful in forming scaffolds from each type of scaffold protein, neutralised type I collagen acetic acid extract, which gels spontaneously on warming from a 4 °C solution to 37 °C; and fibrinogen, enzymatically coagulated with thrombin 37°C. Pore interconnectivity of scaffolds was obtained by controlling the oil:aqueous phase ratio to ≥0.5. Importantly, the formed scaffolds demonstrate a nano-scale fibrous architecture, in addition to the micro-scale pores. The use of an excipient was needed to preserve this structure on lyophilisation and reduce shrinkage. Figure 1a-c shows representative SEM images of each scaffold type.

Pore size of the scaffolds inversely correlated with the surfactant concentration used to create the emulsion (Fig. 1d-f). Previous work showed there was an inverse relationship between surfactant concentration and emulsion droplet diameter (not shown). For scaffolds made from emulsion with 0.1% surfactant, a mean pore size of around 100 μm was obtained, and this decreased to approximately 40 μm with 0.7% surfactant.

### Mechanical properties

The measurement of the tensile properties of each EmDerm scaffold type (Table 1) showed that the ColFbn scaffolds had the highest Young’s modulus and UTS, approximately 1–2 MPa and 12–16 MPa respectively. This is followed by Col scaffolds, which had a Young’s modulus of approximately 1–2 MPa and UTS of 7–9 MPa. Fbn scaffolds had a Young’s modulus of around 1–2 MPa and UTS of 4–5 MPa. Change in porosity of the scaffolds and excipients used did not have a significant impact on mechanical properties of the scaffolds.

**Table 1:** Mechanical strength characterisation of scaffolds (mean ± SD, n = 3)

Scaffold	Young’s modulus (MPa)	Ultimate tensile strength (MPa)
*Collagen scaffolds*		
0.1C	1.95 ± 0.49	8.28 ± 1.62
0.3C	1.45 ± 0.76	7.87 ± 1.45
0.5C	1.71 ± 0.21	7.99 ± 1.02
0.7C	2.14 ± 0.34	8.85 ± 0.97
C-M	1.27 ± 0.48	9.65 ± 2.81
C-P68	1.25 ± 0.74	8.26 ± 1.78
C-PEG	1.70 ± 0.51	8.87 ± 1.24
*Collagen-Fibrin scaffolds*		
0.1CF	2.03 ± 0.81	12.82 ± 3.45
0.3CF	2.43 ± 0.45	14.07 ± 2.67
0.5CF	2.55 ± 0.98	15.15 ± 3.92
0.7CF	2.89 ± 0.72	16.47 ± 5.39
CF-M	1.89 ± 0.58	12.55 ± 3.89
CF-P68	1.46 ± 0.33	13.25 ± 2.66
CF-PEG	1.74 ± 0.76	11.53 ± 1.96
*Fibrin scaffolds*		
0.1F	1.52 ± 0.38	4.98 ± 0.96
0.3F	1.16 ± 0.29	4.81 ± 0.72
0.5F	1.18 ± 0.41	4.77 ± 0.42
0.7F	1.12 ± 0.29	4.62 ± 0.58
F-M	1.26 ± 0.35	4.25 ± 0.63
F-P68	1.48 ± 0.32	4.59 ± 0.22
F-PEG	1.37 ± 0.21	5.13 ± 0.51
F-PVA	2.01 ± 0.11	4.43 ± 0.49

### Hydrolytic degradation of scaffolds

**Fig. 2:**
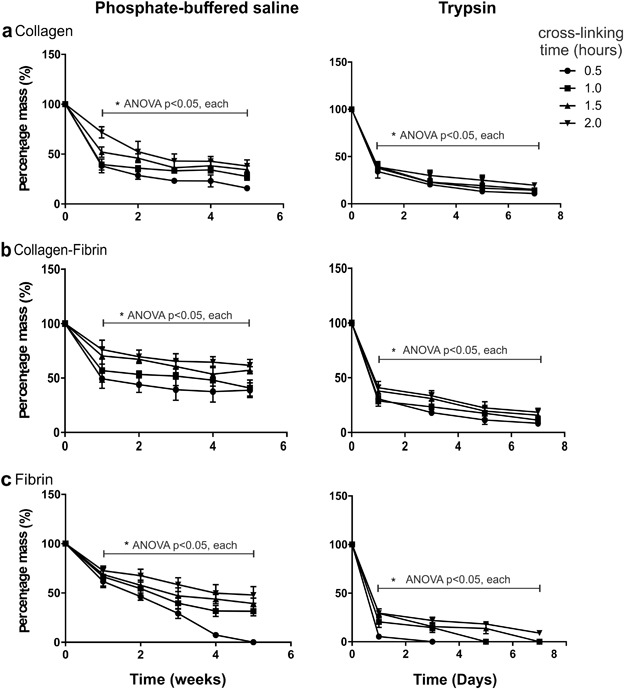
Hydrolytic and enzymatic degradation by mass over a period of time. EmDerm scaffolds formed from: Collagen (**a**), Collagen-Fibrin (**b**) or Fibrin (**c**) with 0.1% surfactant emulsions, cross-linked with EDC/NHS for different durations, after incubation in PBS (up to 5 weeks) or Trypsin (up to 7 days). Data show % reduction of dry weight (mean ± SD, n = 3)

Scaffold degradation by hydrolysis was determined by measuring residual dry weight of scaffolds soaked in PBS for extended periods (Fig. 2a-c). Collagen scaffolds showed a large drop in dry mass of 40–50% over the first week. This is largely due to the dissolution of excipients during the initial hydration of the scaffold. At the end of 5 weeks, all scaffolds retained about 40–60% of their dry mass. Although Col scaffold stability was achieved at the shortest cross-linking time (Fig. 2c), ColFbn and Fbn scaffolds which were cross-linked for a longer duration degraded slower compared to scaffolds which were cross-linked for 30 min (Fig. 2b-c), although this was not statistically significant in this experiment. Notably, Fbn scaffolds which were cross-linked for only 30 min degraded completely by 5 weeks.

### Enzymatic degradation of scaffold

Col and ColFbn scaffolds remained incompletely degraded by Day 7 when incubated in trypsin solution (Fig. 2a, b). All Fbn scaffolds that were cross-linked for less than 2 h were completely degraded within 7 days: Those that were cross-linked for 0.5 h were completely degraded by day 3, followed by those cross-linked for 1 h, on Day 5 and those cross-linked for 1.5 h by day 7 (Fig. 2c). As with the hydrolytic degradation, the mass of each scaffold decreased significantly over the first day. Dissolution of excipients is likely to contribute to the initial drop of scaffold dry mass.

### Cytocompatibility of scaffolds

**Fig. 3:**
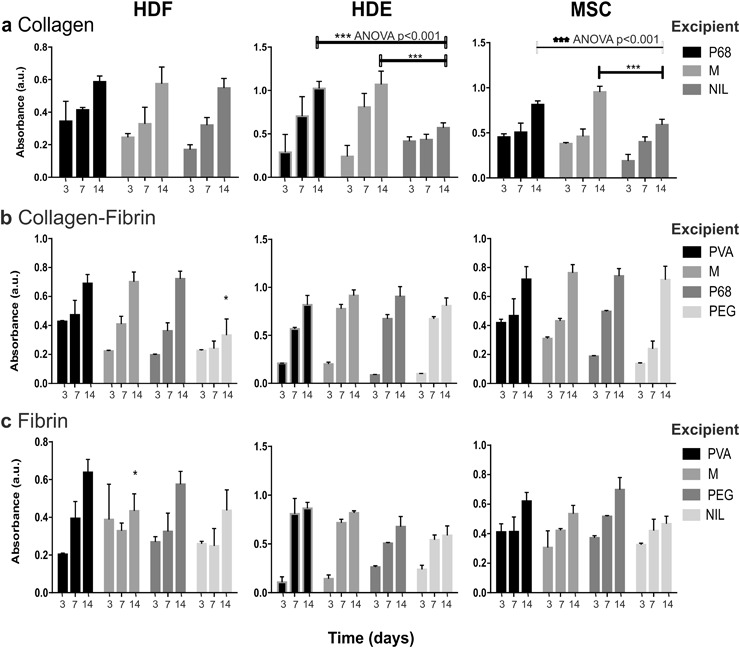
Proliferation of HDF, HDE & MSC in EmDerm scaffolds of collagen (**a**), Collagen-Fibrin (**b**) or Fibrin (**c**) with 0.1% surfactant, prepared with different excipients (PVA, M, P68, PEG) or nil, as described in the methods. Scaffolds were washed, equilibrated and cultured with each cell type, and proliferation was measured by WST-8/PMS reduction on days 3, 7 and 14 (data are mean ± SD, n = 3)

**Fig. 4:**
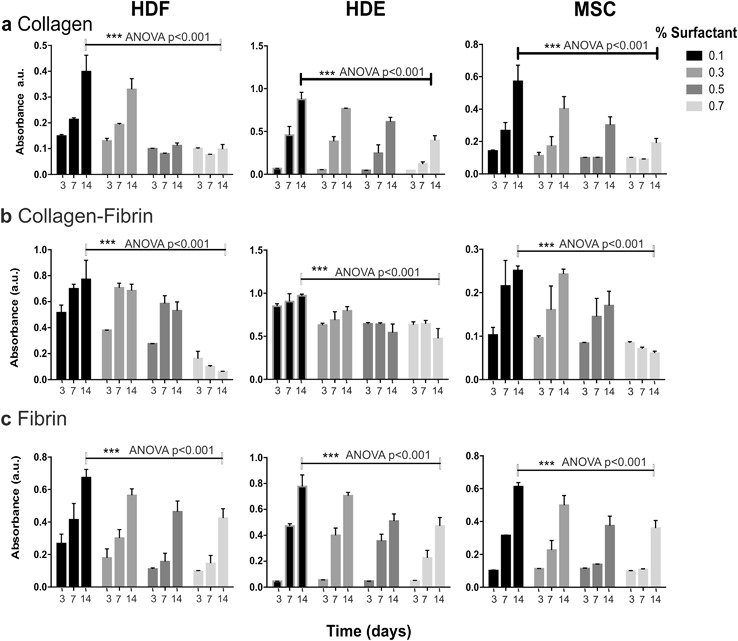
Effect of porosity on in EmDerm collagen (**a**), collagen-fibrin (**b**) and fibrin (**c**) scaffolds achieved by varying surfactant concentration from 0.1 to 0.7% in primary manufacture step on proliferation of cell types (HDF, HDE and MSC) as in Fig. 3 (mean ± SD, n = 3)

**Fig. 5:**
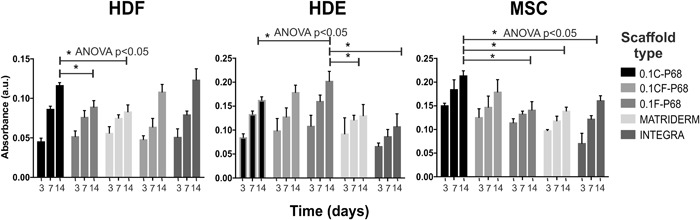
Comparison of the proliferation of HDF, HDE and MSC between the three different types of EmDerm scaffolds made with 0.1% surfactant and P68 excipient, and the commercial reference scaffolds Matriderm™ and Integra™. Cell proliferation was measured as in Fig. 3 (mean ± SD, n = 3)

The assessment of the biocompatibility of EmDerm scaffolds was investigated by measuring the proliferation three cell types central to skin reconstruction, HDF, HDE and MSC. Net proliferation of each cell type occurred over 14 days in each scaffold material (Figs. 3–5). Importantly, there was no significant effect of varying the excipient used, compared to the P68 excipient (Fig. 3). However, it is notable that HDE and MSC proliferation in Col scaffold made without an excipient (C-GEL) was significantly lower than Col scaffolds with excipient (C-P68 and C-M) (Fig. 3a). Also, HDF showed lower proliferation in ColFbn scaffolds with PEG (CF-PEG, Fig. 3b) and F-M (Fig. 3c) scaffolds.

While proliferation was marked in scaffolds with high porosity, there was a general trend for proliferation to be progressively lower with decreased porosity (Fig. 4). This effect was most pronounced for HDF in Col scaffolds at the lowest porosity, corresponding to 0.5 and 0.7% surfactant mix in the emulsion template (Fig. 4a), and in ColFbn scaffolds at the lowest porosity (Fig. 4b).

### Comparison with commercial scaffolds

Significantly greater proliferation of each cell type occurred in each of the high porosity EmDerm scaffolds from 0.1% surfactant than the commercial comparator scaffolds, Matriderm and Integra (Fig. 5). It is notable that the Fbn scaffold 0.1F-P68 supported greatest HDE proliferation, while the Col scaffold 0.1C-P68 promoted the better mesenchymal cell proliferation, both effects being statistically significant (Fig. 5).

### Wide-field microscope imaging of seeded scaffolds

**Fig. 6:**
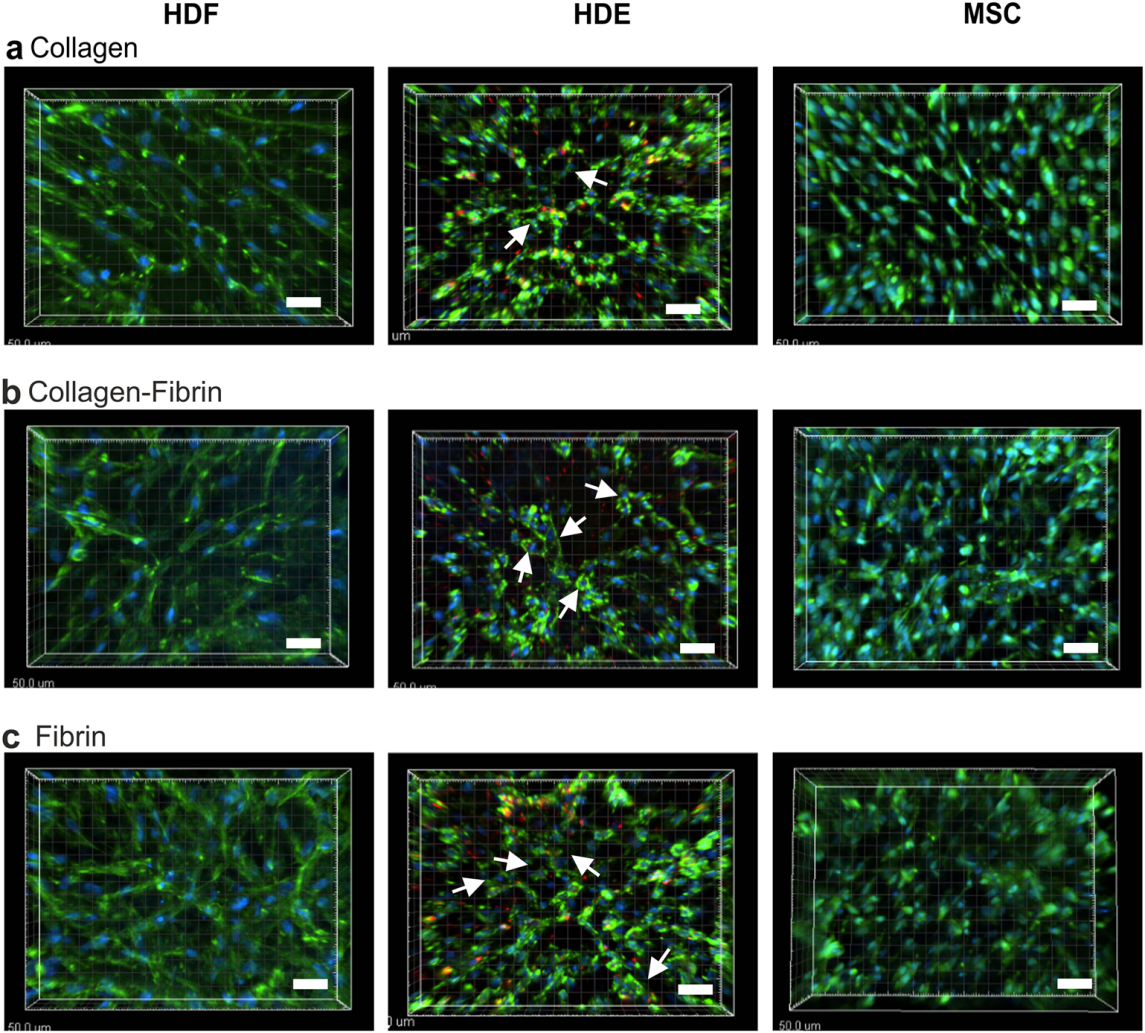
Morphology of HDF, HDE and MSC in the three different types of EmDerm scaffolds made with 0.1% surfactant and P68 excipient, collagen (**a**), collagen-fibrin (**b**) and fibrin (**c**) scaffolds. In HDE-seeded scaffolds, formation of annular structures is apparent (arrows). Scale bars = 50 μm

Based on the Z-stacks obtained, each cell type was found to infiltrate into the scaffold uniformly over the XY plane and Z plane (Fig. 6). Due to limitations in light penetration, cells were only imaged up to a depth of about 100 microns. In particular, cell infiltration into Col scaffolds was observed to a similar extent as for fibrin-containing scaffolds, suggestive of effect of the preserved fibre nanostructure in these scaffolds. Interestingly, the endothelial cells seemed to form ring-like structures when seeded onto EmDerm scaffolds, most notably within ColFbn and Fbn (arrows in Fig. 6, HDE panels). It is also notable that HDE ingress into the EmDerm Col scaffolds, and although show less cytoskeletal spreading, demonstrate some association into annular structures. By contrast, the stromal cell types adopt an elongated spindle-like morphology when seeded on to EmDerm scaffolds, more pronounced with fibroblasts than mesenchymal stem cells (Fig. 6).

## Discussion

In this paper, we demonstrate that an o/w macro-emulsion system can be used successfully as a template for fabricating three-dimensional scaffolds with hierarchical porosity whilst preserving the intrinsic nano-scale protein hydrogel structure. The method enables complex hierarchical structure from the nano-scale to be achieved in a relatively rapid process. Elution of the oil phase by washing can be achieved with several alcohol-based solvents, after cross-linking (data not shown). The resultant EmDerm scaffolds have consistent mechanical properties, are stable in physiological solution, have good biocompatibility and cell conductivity, and support cell proliferation. Effective ingress and proliferation of the main cell types required for dermal reconstruction, HDF, HDE & MSC are shown. Collectively, this suggests that the EmDerm have favourable characteristics as dermal templates for skin regeneration and wound healing.

The characterisation and basic properties of these o/w emulsions have been determined in our laboratory. Structurally, there was no evidence of protein denaturation. When used with acidic extracted type I collagen, which exhibits spontaneous gelation in warming after pH neutralisation, the emulsion does not prevent gelation, and the nanostructure fibrils indicate preservation of the intrinsic structure of the hydrogel. Furthermore, the successful use of emulsion system for fibrin, demonstrates that fibrinogen/thrombin enzymatic coagulation is able to proceed in the mixed phased system, and the resultant fibrin fibril formation is indicative of the native structural self-assembly behaviour of fibrinogen after thrombin cleavage of the fibrinopeptides.

Scaffold porosity is a principle physical determinant of functionality of scaffolds [4, 23, 24]. Control of pore size was achieved by varying the emulsion droplet size through surfactant concentration in the primary manufacture step, at a constant ratio of the aqueous protein phase to the templating oil phase. A direct relationship was found between the surfactant concentration in the primary manufacture step, which determines the mixed phase template droplet diameter, and resultant lyophilised scaffold pore diameter by SEM. This applied over the range of mean pore diameters from around 40 to 100 μm. This suggests that the porosity EmDerm scaffolds can be readily tuned according to needs. A mean pore size between 80 and 100 microns as a suitable target is consistent with optimal migration through scaffolds with this pore size range [25].

The mechanical properties of EmDerm scaffolds suggest that they can be strong enough to be handled physically and do not tear easily. This is imperative as it allows the scaffolds to be positioned and sutured during surgery. With Col scaffolds there was an inverse relationship between the mechanical strength (Young’s modulus and UTS) and porosity, which suggests that some differences in fibril organisation. With Fbn and ColFbn scaffolds, the pore size did not have a marked effect on these parameters

Post-implantation stability of scaffolds is an important aspect of their function, and resorption of tissue regeneration scaffolds allows for new extracellular matrix deposition [25]. Here, we introduced an assay of accelerated proteolytic degradation in order to compare the kinetic profiles of degradation over a relatively short assay period. The cross-linking duration had a direct effect on scaffold stability, demonstrating that this can be controlled. Based on our findings, an incubation time of 1 h for cross-linking should be sufficient to maintain integrity of the scaffolds post-implantation. A shorter duration significantly decreased hydrolytic and proteolytic stability of fibrin-based scaffolds, although the greater stabilisation achieved for collagen scaffolds indicates greater reactivity to the EDC/NHS reagent.

Interestingly, there was a relationship between cell proliferation and porosity, although the surfactant concentration used in the scaffolds’ primary manufacture step is also a variable. The use of concentrations over 0.5% in collagen scaffolds, and 0.7% in collagen-fibrin scaffolds, was associated with marked inhibition of HDF proliferation, possibly suggesting some persistence of surfactant residue during the scaffold processing. However, the other cell types were affected to a lesser extent, which suggests that if surfactant residues persist, the level is close to the threshold to cause cytotoxicity. Additionally, the droplet diameters of emulsions with higher surfactant concentration are much smaller and less ideal for promoting vascularisation. This observation may therefore be useful in establishing a threshold for the safe concentration of the current surfactant in the current manufacture process, and confirms the need to evaluate cytotoxicity. Possibly a more stringent wash process may be required should the higher concentrations be needed. Despite this consideration, the overwhelming evidence of this work is that the EmDerm scaffolds are essentially cytocompatible.

Another aim of this method of manufacture is to create scaffolds with nano-structured architecture, which current commercial scaffolds lack. The nano-fibrous architecture can provide cells with oriented cell adhesion signals and promote migration of cells, as there is an aligned fibre matrix for cells to move along [26]. In this regard, the effect on excipients on preserving the collagen nanostructure of EmDerm scaffolds is notable. By contrast, scaffolds without excipients did not have nano-fibrous walls, rather they were smooth and featureless when imaged under SEM. These findings suggest firstly that collagen is able to undergo structural reorganisation under freeze-drying involving fusion of the fibrillar hydrogel structure into microscale lamellae; secondly that excipient interaction with the protein matrix is a stabilising factor that prevents nano-structure fusion.

The results of the biocompatibility studies with cells relevant to dermal reconstruction are particularly significant. A multi-parameter approach has recently been validated that correlates in vitro cell responses to the clinical properties of dermal biomaterials [27]. The generally greater cell proliferation and migration in the nano-structured EmDerm scaffold may be due to increase in surface area available for cells to adhere to and migrate on, as well as greater permeability to nutrients and oxygen throughout the scaffold [25]. EmDerm scaffolds demonstrate excellent cell penetrance, or conductivity, for different cell types. The present results contrast with previous studies which have shown that collagen as a scaffold material in the commercial scaffolds Integra^TM^ and Matriderm^TM^, and also in decellularised dermal products Alloderm^TM^, Xenoderm^TM^ and Permacol^TM^ and does not support complete cell conductivity, even though the structures have a high degree of interconnected porosity [6, 28]. Thus, the present results suggest that creating a nano-structured form of collagen can significantly increase the cell migration into the scaffold. This could translate to accelerated integration of collagen scaffolds in wound healing settings.

This paper demonstrates that the emulsion templating technique is suitable for creating of a range of nano-structured functional EmDerm scaffolds. The difference in effect of each scaffold material on cell proliferation and morphology suggests that composition may have some cell type specific functional benefits. In particular, the greater proliferation of endothelial cells in collagen-fibrin and fibrin scaffolds than collagen scaffolds is associated with the formation of annular nascent vascular structures. This behaviour, associated with the endothelial interaction with organised fibrin structures, is consistent with other angiogenic properties of fibrin [21, 29–31]. However, the migratory and morphological response of endothelial cells to the nano-structured collagen scaffolds suggests that the nano-structure increases its angiogenic potential. Further examination of the cellular morphological and cell-communication responses will extend our understanding of the role of scaffold nanostructure in influencing cellular behaviour [27] and particularly angiogenesis and vasculogenesis.

## Conclusions

We demonstrate the feasibility of using emulsion templating as a novel method of fabricating micro-porous nano-fibrous protein scaffolds which are unique and easily tuned according to wound healing and tissue regeneration needs. This is a versatile method of templating various protein polymers, including collagen and fibrin, and is amenable to clinical manufacture scaleup. These scaffolds also have excellent cytocompatibility and are able to support various types of cell growth and have excellent potential as dermal or soft tissue substitutes.

## References

[1] Beachley V, Wen X (2010). Polymer nanofibrous structures: fabrication, biofunctionalization, and cell interactions. Prog Polym Sci.

[2] Jones JR, Lee PD, Hench LL (2006). Hierarchical porous materials for tissue engineering. Philos Trans A Math Phys Eng Sci.

[3] Loh QL, Choong C (2013). Three-dimensional scaffolds for tissue engineering applications: role of porosity and pore size. Tissue Eng Part B Rev.

[4] Dagalakis N, Flink J, Stasikelis P, Burke JF, Yannas IV (1980). Design of an artificial skin. Part III. Control of pore structure. J Biomed Mater Res.

[5] Kim TH, Eltohamy M, Kim M, Perez RA, Kim JH, Yun YR (2014). Therapeutic foam scaffolds incorporating biopolymer-shelled mesoporous nanospheres with growth factors. Acta Biomater.

[6] Potter MJ, Banwell P, Baldwin C, Clayton E, Irvine L, Linge C (2008). In vitro optimisation of topical negative pressure regimens for angiogenesis into synthetic dermal replacements. Burns.

[7] Barbetta A, Gumiero A, Pecci R, Bedini R, Dentini M (2009). Gas-in-liquid foam templating as a method for the production of highly porous scaffolds. Biomacromolecules.

[8] Barbetta A, Rizzitelli G, Bedini R, Pecci R, Dentini M. Porous gelatin hydrogels by gas-in-liquid foam templating. Soft Matter. 2010;6. 10.1039/b920049e.

[9] Schacht K, Vogt J, Scheibel T (2016). Foams made of engineered recombinant spider silk proteins as 3D scaffolds for cell growth. ACS Biomater Sci Eng.

[10] Vrana NE, Builles N, Kocak H, Gulay P, Justin V, Malbouyres M (2007). EDC/NHS cross-linked collagen foams as scaffolds for artificial corneal stroma. J Biomater Sci Polym Ed.

[11] Zhu N, Chen X. Biofabrication of tissue scaffolds. In: Pignatello R, editor. Advances in biomaterials science and biomedical applications. InTech: London, UK; 2013. pp. 315–28. 10.5772/54125.

[12] Cameron NR (2005). High internal phase emulsion templating as a route to well-defined porous polymers. Polymer.

[13] Franks GV, Moss B, Phelan D (2006). Chitosan tissue scaffolds by emulsion templating. J Biomater Sci Polym Edn.

[14] Hulda-Chambi CG, Grosso C, editors. Production and characterization of multicomponent films based on polysaccharides, gelatin and lipids: Effect of surfactants addition. International Congress on Engineering and Food; 2011 22-26/5/2011; Cosmosware, Athens, Greece: Elsevier Procedia.

[15] Dalgleish DG (1997). Adsorption of protein and the stability of emulsions. Trends Food Sci Technol.

[16] Chen J, Dickinson E, Iveson G (1993). Interfacial interactions, competitive adsorption and emulsion stability. Food Struct.

[17] Dickinson E (1998). Proteins at interfaces and in emulsions stability, rheology and interactions. J Chem Soc, Faraday Trans.

[18] Kim H, Decker E, McClements D (2002). Impact of protein surface denaturation on droplet flocculation in hexadecane oil-in-water emulsions stabilized by β-lactoglobulin. J Agric Food Chem.

[19] McClements DJ (2004). Protein-stabilized emulsions. Curr Opin Colloid Interface Sci.

[20] Pham C, Greenwood J, Cleland H, Woodruff P, Maddern G (2007). Bioengineered skin substitutes for the management of burns: a systematic review. Burns.

[21] Ahmed TA, Dare EV, Hincke M (2008). Fibrin: a versatile scaffold for tissue engineering applications. Tissue Eng Part B Rev.

[22] Shepherd DV, Shepherd JH, Ghose S, Kew SJ, Cameron RE, Best SM. The process of EDC-NHS cross-linking of reconstituted collagen fibres increases collagen fibrillar order and alignment. APL Mater. 2015;3. 10.1063/1.4900887. 10.1063/1.4900887 PMC4262854 25506518

[23] Murphy CM, Haugh MG, O’Brien FJ (2010). The effect of mean pore size on cell attachment, proliferation and migration in collagen–glycosaminoglycan scaffolds for bone tissue engineering. Biomaterials.

[24] O’Brien FJ (2011). Biomaterials & scaffolds for tissue engineering. Mater Today.

[25] Velasco MA, Narvaez-Tovar CA, Garzon-Alvarado DA (2015). Design, materials, and mechanobiology of biodegradable scaffolds for bone tissue engineering. Biomed Res Int.

[26] Santos MI, Tuzlakoglu K, Fuchs S, Gomes ME, Peters K, Unger RE (2008). Endothelial cell colonization and angiogenic potential of combined nano- and micro-fibrous scaffolds for bone tissue engineering. Biomaterials.

[27] Garcia-Gareta E, Ravindran N, Sharma V, Samizadeh S, Dye JF (2013). A novel multiparameter in vitro model of three-dimensional cell ingress into scaffolds for dermal reconstruction to predict in vivo outcome. Biores Open Access.

[28] Wahl EA, Fierro FA, Peavy TR, Hopfner U, Dye JF, Machens HG (2015). In vitro evaluation of scaffolds for the delivery of mesenchymal stem cells to wounds. Biomed Res Int.

[29] Caiado F, Carvalho T, Silva F, Castro C, Clode N, Dye JF (2011). The role of fibrin E on the modulation of endothelial progenitors adhesion, differentiation and angiogenic growth factor production and the promotion of wound healing. Biomaterials.

[30] Shaikh FM, Callanan A, Kavanagh EG, Burke PE, Grace PA, McGloughlin TM (2008). Fibrin: a natural biodegradable scaffold in vascular tissue engineering. Cells Tissues Organs.

[31] Potter MJ, Linge C, Cussons P, Dye JF, Sanders R (2006). An investigation to optimize angiogenesis within potential dermal replacements. Plast Reconstr Surg.

